# Characteristics of Older Adults With Domestic Squalor in Japan: A Cross‐Sectional Study

**DOI:** 10.1111/psyg.70150

**Published:** 2026-02-25

**Authors:** Daiki Taomoto, Hideki Kanemoto, Takashi Suehiro, Yuto Satake, Naomi Nakamuta, Maki Hotta, Maki Suzuki, Kenji Yoshiyama, Toyoko Miyoshi, Kazue Kashiwagi, Kazue Shigenobu, Mamoru Hashimoto, Manabu Ikeda

**Affiliations:** ^1^ Department of Psychiatry The University of Osaka Graduate School of Medicine Osaka Japan; ^2^ Health and Counselling Center, the University of Osaka Osaka Japan; ^3^ Department of Behavioral Neurology and Neuropsychiatry United Graduate School of Child Development, the University of Osaka Osaka Japan; ^4^ Asakayama General Hospital Osaka Japan; ^5^ Department of Neuropsychiatry Kindai University Faculty of Medicine Osaka Japan

**Keywords:** dementia, Diogenes syndrome, hoarding symptom, initial‐phase intensive support team for dementia, self‐neglect

## Abstract

**Background:**

Domestic squalor (DS) is related to physical problems, living alone, family and neighbourhood problems and mental illnesses, especially dementia. However, methods to detect and treat older adults with DS remain unclear. We aimed to clarify the methods of appropriate intervention for older adults with DS.

**Methods:**

The Initial‐phase Intensive Support Team for Dementia (IPIST) is a multidisciplinary outreach team in Japan that provides intensive support to people living at home with suspected dementia. We distributed a questionnaire to 50 IPISTs, specifically requesting the submission of ‘two complex cases that were difficult to manage’ regarding medical or long‐term care. The questionnaire included sociodemographic characteristics, diagnosis, referral sources, and reasons for complexity. We compared cases with and without DS (DS+ vs. DS‐), and DS cases living alone and those living with family (DS+ living alone vs. DS+ living with family).

**Results:**

We received responses from 33 IPISTs and collected data from 70 complex cases. DS was selected as the reason for complexity in 24 cases, 14 of which were people living alone. Significantly fewer DS+ cases were referred by family (8.3% vs. 54.3%), while significantly more were referred by others (87.5% vs. 41.3%). The most common referral sources were neighbours and social workers. DS+ cases had significantly higher rates of self‐neglect (87.5% vs. 13.0%). Those living alone were significantly younger (median (interquartile range): 78.5 (76.8–82.8) years) than those living with family (85.0 (78.8–89.8) years). Family members living with DS+ cases also had issues, including mental illness or maltreatment.

**Conclusions:**

In this study sample, many older adults with DS exhibited self‐neglect and had limited family support. Our findings suggest that a comprehensive approach to both older adults with DS and their families is crucial for appropriate intervention. Therefore, IPISTs that have a multidisciplinary team with an outreach function may be effective.

## 1 Introduction

Domestic squalor (DS), which is called ‘gomi‐yashiki’ in Japanese, is defined as a living environment that is ‘so unclean, messy and unhygienic that people of similar culture and background would consider extensive clearing and cleaning to be essential’ [1]. Past studies have reported an estimated prevalence of DS between 0.05%–0.12% in older adults [2, 3, 4, 5] and 0.03%–0.85% across all ages [3, 6]. DS has also been described as senile breakdown [2], and Diogenes syndrome [7].

Self‐neglect is a concept that overlaps with DS, and the latter may result from the former [8]. Self‐neglect includes all forms of neglect of the self, and not solely environmental neglect. Therefore, it may include people who live in clean environments but neglect personal hygiene, food, or medical care. Consistent with current clinical understanding, in this study, DS is conceptualized not as an independent diagnostic category, but as a severe clinical phenotype characterized by extreme environmental neglect. Although DS is not considered a distinct disorder in current classification systems, past studies have reported relationships between DS and mental illnesses, the most common of which is dementia [3, 4, 5, 9, 10, 11].

In addition, past studies have reported relationships between DS and physical problems [2, 7, 11, 12, 13, 14], living alone [7, 15, 16], and problems for the person's family and neighbours [17, 18]. Expanding the perspective to the broader concept of self‐neglect, recent community‐based studies in the Mediterranean and Southwest Asia have highlighted significant associations with depression, frailty, and limited social support [19, 20, 21]. These findings suggest that shared vulnerability factors exist across different sociocultural contexts, relevant to both self‐neglect and DS.

Regardless of whether DS arises as an independent condition or a manifestation of neurocognitive disorders, it presents specific management challenges—such as refusal of services and environmental hazards—that require distinct intervention strategies beyond standard dementia care. Therefore, appropriate intervention for DS is crucial; however, how to detect and approach older people with DS is unclear because past studies related to DS in Japan are limited [11, 14, 22].

In Japan, the Initial‐phase Intensive Support Team for Dementia (IPIST) works with many older people with DS. IPIST is a multidisciplinary outreach team that conducts extensive initial assessment and support for people living at home with suspected dementia in Japan [23, 24]. Of IPIST cases, 40% are ‘complex cases’ that are extremely challenging to approach or link to medical or long‐term care [25]. Our previous nationwide survey identified the primary drivers of this complexity as ‘refusal of public support, refusal to see a doctor, and refusal of IPISTs' visiting’, ‘behavioral and psychological symptoms of dementia (BPSD)’, ‘complications of mental illness’, ‘mental illness in the persons living with them’ and ‘trash house’. Trash house accounted for 4.3% of the causes of these difficult situations [26].

This study aimed to clarify the methods of appropriate intervention for older people with DS by investigating its characteristics. To this end, we analysed questionnaire responses regarding these cases, focusing on DS, self‐neglect, hoarding symptoms, physical problems and living alone.

## 2 Methods

This study was conducted in accordance with the World Medical Association's Declaration of Helsinki (2008). This study was approved by the Ethics Committees of Osaka University Hospital (Study Number 21353 (T1), Suita, Japan).

### 2.1 Participants and Procedure

This was a cross‐sectional questionnaire study. The Japanese government initiated a model project of IPIST in 2013 and subsequently required all municipalities, including cities, towns, and villages, to set up at least one team, which was completed in September 2019. Each IPIST included at least one physician and two non‐physician professionals, such as public health nurses and home care workers. Individuals who are eligible for IPIST include those living at home who are aged ≥ 40 years, who are suspected of having dementia or who have dementia and fit into one of the following categories: (1) those who are not using medical or long‐term care services or those who have discontinued the services they were using, and (2) those who are using medical and long‐term care services but are difficult to treat because of severe BPSD.

In our previous nationwide survey on IPIST's complex cases, a questionnaire was distributed electronically to each IPIST through all 1741 municipalities in Japan and responses were collected from 14 January to 8 March 2021 [26]. From this survey, we extracted 50 IPISTs that had reported experience of working with many complex cases, adopting a purposive sampling approach to target teams with rich experience in difficult interventions. In the present study, we electronically distributed a questionnaire about the complex cases to the 50 IPISTs and collected responses from 11 January to 18 February 2022.

### 2.2 Questionnaire

Based on our nationwide survey in 2021 [26], D.T., K.S., M.H. and M.I. discussed and developed a questionnaire to survey complex cases that the IPIST worked with and created a manual for complex cases (Figure S1). To clarify the characteristics of challenging interventions, we utilized a purposive sampling strategy for case selection; specifically, we asked each IPIST to submit ‘two complex cases that were difficult to manage’ regarding medical or long‐term care [25]. In large municipalities, independent IPISTs are established for specific jurisdictional districts (covering one or multiple administrative wards). In such cases, we treated each district team as a distinct unit for data collection, maintaining the limit of two cases per independent team.

The questionnaire collected information about complex cases including age, sex, living condition (living alone or living with family), diagnosis, history of present illness, referral sources and reasons for the complexity. Referral sources were selected from the following: family, self, medical institution, care manager, welfare commissioner, neighbour, or other. Reasons for the complexity were one or more of the following: reasons for the older persons receiving IPIST support (hallucinations/delusion, irritability/aggression, symptoms of disease, violence, drinking, self‐care, driving, taking medicine and financial management), caregivers' reasons (absence, old age, exhaustion, physical disease, mental disease, maltreatment against cases, lack of understanding and poverty), social reasons (trash house, problems with fire, problems with neighbour, and police intervention) and other reasons (free‐text comment). History of present illness was used to assess ‘hospitalization owing to the physical illness’ and ‘living in a poor residential environment’. We provide more details on these terms in the sections mentioned later. The characteristics of their family were assessed by reasons for complexity and history of present illness.

### 2.3 Self‐Neglect

We defined self‐neglect when the individuals lived in a poor residential environment and lacked self‐care. This was based on a common definition of self‐neglect in Japan as ‘abandoning either or all of personal hygiene, cleanliness/arrangement of the residential environment, or health action,’ with the following two main elements: ‘lack of self‐care’ and ‘poor residential environment’ [27]. If a trash house was selected as the reason for complexity in the questionnaire, or poor residential environment was described in the history of present illness, the individual was considered to live in a poor residential environment. If problems with self‐care were selected as the reason for complexity in the questionnaire, the individual was considered lacking in self‐care.

### 2.4 Clutter Image Rating

We defined the cases with DS when trash house was selected as the reason for the complexity. Of the cases with DS, we additionally examined clutter image rating (CIR) [28], which was developed to evaluate the severity of hoarding symptoms, to aid in evaluation of the severity of DS in this study. The CIR is a visual assessment consisting of nine pictures showing gradual clutter in each of three rooms (kitchen, living room and bedroom), and the individuals choose pictures that reflect the states of their homes most accurately. In this study, we asked IPIST members to choose pictures most closely resembling the states of the individuals' homes. The chosen pictures were converted into points, and the CIR score was calculated by averaging the total points of the three rooms. When CIR was collected in only two rooms, its score was calculated by averaging the total points of the two rooms. The cut‐off score for clinical‐level hoarding is 4 points [28].

### 2.5 Analyses

We compared sociodemographic characteristics, diagnosis, referral source, self‐neglect, and hospitalization due to physical illness between the individuals with and without DS (DS+ vs. DS‐) and between those with DS who lived alone and those who lived with family (DS+ living alone vs. DS+ living with family). We compared CIR only between DS+ living alone and DS+ living with family.

For the above comparisons, we used the Mann–Whitney U test for the continuous and ordinal variables and Fisher's exact test for the nominal variables. All analyses were performed using IBM SPSS Statistics for Windows, Version 27.0 (IBM Corp., Armonk, NY, USA). The level of statistical significance was set at two‐tailed *p* < 0.05.

## 3 Results

### 3.1 All Cases

We received responses from 33 municipalities (questionnaire response rate: 66%). In total, 70 cases were collected: two municipalities submitted one case, 29 municipalities submitted two cases, one municipality submitted four cases, and one municipality submitted six cases. However, the submissions of four and six cases were from designated cities, representing aggregated data from two and three independent teams operating in distinct districts, respectively (each contributing two cases). Therefore, the protocol of collecting two cases per independent team was effectively maintained.

The age (median (interquartile range)) of these individuals was 80.0 (75.0–85.0) years, and the male‐to‐female ratio was 37.1%:62.9%. Of them, 51.4% lived alone. Mental illness was diagnosed in 88.6% of the individuals (62/70), among whom dementia was the most common (57/70; 81.4%); with Alzheimer's disease (AD) being the most common subtype of dementia (31/70; 44.3%). The most common referral source was family, with 38.6% of cases being referred in this manner. Of the individuals in these cases, 47.1% lived in a poor residential environment, 55.7% lacked self‐care, and 18.6% were hospitalized due to physical illness during the intervention of IPIST.

### 3.2 Comparison of Cases With and Without Domestic Squalor

DS was selected as the reason for complex cases in 24 (34.3%) (DS+) of all 70 cases (Table 1). There were no significant differences between DS+ and DS‐ groups in terms of age, sex, living condition, diagnosis, and hospitalization due to physical illness. The proportion of individuals referred by the family was significantly lower in DS+ than in DS‐ (8.3% vs. 54.3%, *p* < 0.001) although the proportion of those living with their families was similar. Cases referred by others were significantly more common in DS+ cases than in DS‐ cases (87.5% vs. 41.3%). In DS+ cases, the most common referral sources were neighbours and welfare commissioners (20.8% each). In contrast, in DS‐ cases, the most common referral source was family. Self‐neglect was significantly more common in DS+ than in DS‐ individuals (87.5% vs. 13.0%, *p* < 0.001). All 100% of DS+ individuals lived in poor residential environments. For example, their home was unsanitary because garbage was piled up, excrement was not cleaned, and this garbage and excrement produced a bad odour. Of these DS+ individuals, 87.5% lacked self‐care. Details about each DS+ case, such as age, sex, diagnosis, living condition, characteristic of family, CIR, and details about DS, are shown in Table 2.

**TABLE 1:** Comparison of cases with and without domestic squalor.

	DS+	DS–	*p*
Number of cases	24 (34.3%)	46 (65.7%)	
Age (years)	80.5 (77.3–85.0)	79.5 (73.8–84.0)	0.382
Sex, female	14 (58.3%)	30 (65.2%)	0.610
Living condition, living alone	14 (58.3%)	22 (47.8%)	0.457
Diagnosis			
Dementia	22 (91.7%)	35 (76.1%)	0.194
AD	10 (41.7%)	21 (45.7%)	
Frontotemporal dementia	3 (12.5%)	2 (4.3%)	
AD with cerebrovascular disease	2 (8.3%)	2 (4.3%)	
Dementia with Lewy bodies	2 (8.3%)	2 (4.3%)	
Vascular dementia	0 (0%)	3 (6.5%)	
Other types of dementia	1 (4.2%)	1 (2.2%)	
Unspecified dementia	4 (16.7%)	7 (15.2%)	
Mild cognitive impairment	0 (0%)	1 (2.2%)	1.000
Alcoholism	0 (0%)	1 (2.2%)	1.000
Epilepsy	0 (0%)	1 (2.2%)	1.000
Psychiatric disorders	1 (4.2%)	3 (6.5%)	1.000
Intellectual disabilities	0 (0%)	1 (2.2%)	1.000
No diagnosis	1 (4.2%)	7 (15.2%)	0.249
Referral source (Family: Self: Other)	2^a^: 1: 21^b^	25^a^: 2: 19^b^	< 0.001
Self‐neglect	21 (87.5%)	6 (13.0%)	< 0.001
Poor residential environment	24 (100%)	9 (19.6%)	< 0.001
Lack of self‐care	21 (87.5%)	18 (39.1%)	< 0.001
Hospitalization due to physical illness	6 (25.0%)	7 (15.2%)	0.346

*Note:* Data are presented as median (interquartile range) or number (%).

Abbreviations: AD, Alzheimer's disease; DS, domestic squalor; NA, not applicable.

^a^ With DS < without DS (in post hoc test).

^b^ With DS > without DS (in post hoc test). Mann–Whitney U test was used for the continuous variables. Fisher's exact test was used for the nominal variables. The level of statistical significance was set at two‐tailed *p* < 0.05.

**TABLE 2:** Detailed characteristics of DS+ cases.

Case no	Age (years)/Sex	Diagnosis	Living condition	Characteristic of family	CIR kitchen/bedroom/living room	Detail about DS
1	60s/Male	FTD	Living alone		3/4/4	The dog is chewing on his bed and his room is full of cotton from his bed.
2	60s/Male	Dementia (other type)	Living alone		3/2/3	
3	70s/Male	VaD + AD	Living alone		4/4/4	The environment is unsanitary. Excrement has accumulated in the room. There is rotten food in the refrigerator and flies are flying around.
4	70s/Male	AD	Living alone		8/5/5	His home and garden are full of garbage. His bed is incontinent and unsanitary. There is a burnt mark on the furniture, and the risk of fire is high.
5	70s/Male	Schizophrenia	Living alone		4/6/4	
6	70s/ Female	AD	Living alone		5/NA/9	
7	70s/Female	Dementia	Living alone		4/NA/4	Garbage is cluttered both indoors and outdoors.
8	70s/Female	AD	Living alone		3/3/3	There is a lot of garbage and dog hair in her room.
9	80s/Male	AD	Living alone		7/4/7	His room shows evidence of DS, and flies are flying around in the summer.
10	80s/Male	AD	Living alone		4/4/4	There is plenty of rotten foods, and flies are flying around.
11	80s/ Female	AD	Living alone		NA	Valuables such as passbooks, insurance cards, and stamps are found in a pile of garbage in the room.
12	80s/Female	AD	Living alone		5/3/5	After her family had cleaned up her room, her room has been filled with a garbage in a week.
13	80s/Female	AD	Living alone		4/4/4	Because the toilet cannot not be used owing to clogging by excrement, she is using the toilet in the nearby supermarket during the day and a diaper at night.
14	80s/Female	Dementia	Living alone		3/3/3	She has 15 cats.
15	70s/Male	FTD	Living with wife	The individual commits violence against his wife.	3/4/4	His refrigerator is unsanitary, because it is crammed with a lot of foods, which are rotten and smell bad.
16	70s/Female	AD	Living with husband	Her husband has a tendency to hoard.	NA	Her room is cluttered, leaving no space to put her feet. Her medicine is scattered on the floor.
17	80s/Male	FTD	Living with a child	His child has physical disability and psychiatric disease.	4/NA/5	His body, clothes and living condition are unsanitary.
18	80s/Male	Dementia	Living with wife	His wife wears unclean clothes with bad odour.	5/7/6	Excrement and garbage are piled up. There is rat manure in the room, the tatami mats are rotten, and the floor is damaged.
19	80s/Female	NA	Living with a child	Her adult child mistreats her. Her child's room is unsanitary and cluttered.	3/4/3	There is cluttered garbage, leaving no space to put her feet. Her house is like a ruin because there is an accumulation of garbage bags. Birds and insects are flying in her room.
20	80s/Female	VaD + AD	Living with a child	Her adult child mistreats her. Her child strongly refuses medical and welfare services.	7/7/7	
21	80s/Female	DLB	Living with husband	Her husband has a schizophrenia.	3/4/7	
22	80s/Female	Dementia	Living with a grandchild	Her grandchild is a high school student.	4/9/4	She has not been successful at tidying her room. Her kitchen, bathroom and toilet appeared to have been neglected for many years. Her room is cluttered, and flies are flying in her room. It is full of bad odour from cats.
23	90s/Female	DLB	Living with a child	Her child has intellectual and physical disability.	2/3/3	Her bed is soiled with stool.
24	90s/Female	AD	Living with a child	Her child mistreats the case.	8/7/8	Neighbours complain about bad odour from the garbage.

Abbreviations: AD, Alzheimer's Disease; CIR, clutter image rating; DLB, dementia with Lewy bodies; DS, domestic squalor; FTD, frontotemporal dementia; NA, not applicable; VaD, vascular dementia.

### 3.3 Comparison of Cases With Domestic Squalor Who Lived Alone With Those Who Lived With Their Family

Of 24 DS+ individuals, 14 (58.3%) lived alone (DS+ living alone), and 10 (41.7%) lived with their family (DS+ living with family). DS+ individuals living alone were significantly younger than those living with family (78.5 (76.8–82.8) years vs. 85.0 (78.8–89.8) years, *p* = 0.048, Table 3). There was no significant difference between the two groups in terms of sex, diagnosis, referral sources, self‐neglect and hospitalisation due to physical illness.

**TABLE 3:** Comparison of cases with domestic squalor who lived alone to those who lived with family.

	DS+ living alone	DS+ living with family	*p*
Number of cases	14 (58.3%)	10 (41.7%)	
Age (years)	78.5 (76.8–82.8)	85.0 (78.8–89.8)	0.048
Sex, female	7 (50.0%)	7 (70.0%)	0.421
Diagnosis			
Dementia	13 (92.9%)	9 (90.0%)	1.000
AD	8 (57.1%)	2 (20.0%)	
Frontotemporal dementia	1 (7.1%)	2 (20.0%)	
AD with cerebrovascular disease	1 (7.1%)	1 (10.0%)	
Dementia with Lewy bodies	0 (0%)	2 (20.0%)	
Other types of dementia	1 (7.1%)	0 (0%)	
Unspecified dementia	2 (14.3%)	2 (20.0%)	
Psychiatric disorders	1 (7.1%)	0 (0%)	1.000
No diagnosis	0 (0%)	1 (10.0%)	0.417
Referral source (Family: Self: Other)	0: 1: 13	2: 0: 8	0.163
Clutter image rating			
Average	4.0 (3.3–5.3)	4.7 (3.5–6.5)	0.393
Kitchen	4.0 (3.0–5.0)	4.0 (3.0–6.0)	0.744
Bedroom	4.0 (3.0–4.0)	5.5 (4.0–7.0)	0.075
Living room	4.0 (3.5–5.0)	5.0 (3.5–7.0)	0.431
Self‐neglect	13 (92.9%)	8 (80.0%)	0.550
Poor residential environment	14 (100%)	10 (100%)	NA
Lack of self‐care	13 (92.9%)	8 (80.0%)	0.550
Hospitalisation due to physical illness	5 (35.7%)	1 (10.0%)	0.341

*Note:* Data are presented as median (interquartile range) or number (%). Mann–Whitney U test is used for the continuous and ordinal variables. Fisher's exact test is used for the nominal variables. The level of statistical significance is set at two‐tailed *p* < 0.05.

Abbreviations: AD, Alzheimer's disease; DS, domestic squalor; NA, not applicable.

Among the 10 individuals in the DS+ group living with family, 5 lived with their children, 4 with a partner, and 1 with a grandchild. In most cases, they were not supported because of family problems such as mental illness, maltreatment against cases, lack of self‐care, and young carers. In one case, the individual was not supported because of the violence towards the family.

Among the 24 DS+ individuals, CIR could not be assessed in two cases and the bedroom could not be rated in three. In DS+ cases, the CIR scores for average, kitchen, bedroom, and living room were above the original cut‐off of 4 points (4.2 (3.6–6.0), 4.0 (3.0–5.0), 4.0 (3.0–6.0) and 4.0 (3.8–6.3), respectively). There was no significant difference in the CIR scores between DS+ living alone and DS+ living with family.

## 4 Discussion

The present survey indicated that DS was one of the major reasons for ‘complex cases’ that are extremely difficult to approach or connect to medical or long‐term care among older people in Japan. We found that DS+ individuals had self‐neglect and were not referred by family, whereas family was the most common referral source among DS‐ individuals. Moreover, we found no significant difference between DS+ cases living alone and those living with family, except for age. We considered that DS could have occurred even with a live‐in family member, as the family member also had mental health problems such as schizophrenia, developmental disorders, and hoarding symptoms. The observation that individuals with DS who live with family often cohabit with relatives experiencing their own psychosocial difficulties is particularly noteworthy. This finding suggests that effective interventions should target not only the older person with DS but also the broader family system.

In the present study, DS was selected as the reason for complexity in 34.4% of cases (24/70). In our previous study, trash house accounted for 4.3% of the causes of difficulties in IPISTs' complex cases [26]. Since we asked each IPIST to submit only two complex cases out of many complex cases in the present study, it is possible that the most difficult cases were selected, which may have resulted in a higher proportion of DS in the present study than in our previous study. This suggests that DS is less common in complex cases in people with dementia, but intervention for DS is more likely to be difficult. Past studies reported that DS was related to mental illnesses, most commonly dementia [3, 4, 5, 9, 10, 11], such as frontotemporal dementia (FTD) or more advanced stages of AD [29]. Dementia, particularly AD, is the most common cause of DS in Japan and Hong Kong [9, 11]. In the present study, 91.7% of DS+ individuals had a diagnosis of dementia, with AD being the most common, followed by FTD. These results are consistent with past studies. However, recognising DS solely as a symptom of dementia risks underestimating the specific management challenges it presents, such as environmental hazards, fire risks, and neighbourhood conflicts. Regardless of the underlying pathology, identifying DS as a distinct clinical phenotype is essential for implementing targeted interventions—such as environmental clearing and legal coordination—that go beyond standard dementia care.

Regarding associated factors, past studies have reported relationships between DS and physical problems [2, 7, 11, 12, 13, 14] as well as problems with the person's family and neighbours [17, 18]. Our findings align with recent epidemiological studies from other regions, including the Mediterranean and Southwest Asia [19, 20, 21]. These community‐based studies have similarly highlighted strong associations between self‐neglect and conditions, such as depression, frailty and social isolation, reinforcing the global nature of these clinical challenges. To improve these problems, appropriate intervention for DS based on the clinical assessment is crucial.

Among young people, one of the causal disorders for DS is a hoarding disorder (HD), which is a disease concept newly presented in the Diagnostic and Statistical Manual of Mental Disorders, Fifth Edition [30]. However, in the present study, HD was not extracted as a factor associated with DS. Typically, HD starts in the early teenage years, and the clinical course of HD is chronic, and patients rarely show spontaneous remission [30]. Therefore, older people with HD may not be referred to IPIST because they usually maintain activities of daily living, such as self‐care, or it may be difficult to diagnose that cases with young‐onset HD have complicated dementia at an older age since it is difficult to assess hoarding symptoms in younger age by informants. In addition, HD may be underdiagnosed in the present study because HD, especially late‐onset HD, is a newly presented concept and therefore not well known to the survey respondents. Cath et al. reported that the prevalence of provisional HD diagnosis was steadily increased by 20% every 5 years of age [31]. Dozier et al. reported that approximately one‐fourth of older adults with HD reported a possible onset after the age of 40 [32]. We should be aware of the possible involvement of HD in DS in older people. Patients with HD consider the items, which seem unnecessary to other people, as useful or beautiful, or they have a strong attachment to these items [33]. Therefore, they show strong resistance to disposing of things to eliminate DS. In contrast, most patients with FTD show little interest in the accumulated items and can allow others to discard them, without experiencing distress [34]. Accordingly, the intervention for patients with HD would be different from that for patients with hoarding symptoms due to FTD. We should carefully discuss with patients with HD how to treat the items that seem unnecessary to other people.

In the present study, the proportion of patients in the DS+ group who were self‐neglecting was significantly higher than that in the DS‐ group. Self‐neglecting people refuse or fail to provide themselves with adequate food, water, clothing, shelter, personal hygiene, medication, and safety [35]; some of these actions may also occur in cases of DS [8]. Self‐neglect has been reported to increase the risk of death in older adults independently of chronic physical diseases [36]. This suggests the importance of early intervention for DS. However, self‐neglect makes it difficult for them to seek support from others on their own, making a proactive approach from others important for early intervention. This is probably reflected in the fact that in the present study, a significantly higher proportion of the DS+ group than the DS‐ group requested IPIST interventions from people other than themselves or their family members, with only 4.2% of the requests coming from the person themselves. This is consistent with past studies that self‐referral in patients with DS ranged from 0% to 5% [3, 9, 37] and that the most common referral sources in the patients with DS were social services [3], neighbours from other households [9], and inpatient social workers [37].

The most common referral source depended on the local system and was not the family. These results are consistent with past studies [3, 9, 37]. To ensure early intervention for older people with DS, an early referral from neighbours and medical and welfare experts is necessary. In addition, IPIST with outreach function may be useful for older people with DS, who have few self‐referrals. In past studies, the majority of the individuals with DS lived alone [7, 15, 16], and living alone has been considered one of the risk factors for DS. However, the findings of the present study indicated that more than 40% of those with DS lived with their family (41.7%). This discrepancy may be due to the purposive sampling strategy of the present study, which collected only two ‘difficult’ complex cases from each IPIST. The family members who lived with individuals that belonged to DS+ cases collected in the present study had some problems, such as mental illness; interventions may be more challenging when both the patient and cohabiting family members have mental health issues than with those with DS living alone. Therefore, it is possible that DS+ cases living with family members were prioritized in the present study compared with previous studies. Consequently, it is crucial to consider how DS is affected by the characteristics of both the dementia patients themselves and family cohabitants with mental illness and other problems, and to target interventions not only at the patients themselves but also at the broader family system. More detailed study of family members living with those with DS, as well as the person with dementia, may be needed to inform appropriate interventions for DS in older people with dementia.

We examined CIR [28] to aid in the evaluation of the severity of DS. In the present study, the CIR score of DS+ for average, kitchen, bedroom and living room was above the original cut‐off of 4 points (4.2 (3.6–6.0), 4.0 (3.0–5.0), 4.0 (3.0–6.0) and 4.0 (3.8–6.3), respectively). In a past study on older adults with HD, the CIR score for average, kitchen, bedroom, and living room was 4.0 ± 1.8, 3.6 ± 1.9, 4.1 ± 2.2 and 4.3 ± 2.1, respectively [38], which was slightly lower than that in the present study. This difference may be caused by the difference in referral source, clinical diagnosis and selection bias. The individuals in the present study were referred by others, whereas those in the past study were self‐referred [38]. All individuals in the past study were diagnosed with HD; however, none of the participants in the present study had a diagnosis of HD. These findings suggest that older adults with DS, even those primarily diagnosed with dementia rather than HD, present with severe environmental hoarding symptoms that require objective assessment. CIR may be able to assess several symptoms even in older people with DS other than HD. To the best of our knowledge, the present study is the first to evaluate the severity of hoarding symptoms in older people with DS other than HD, using CIR, highlighting the value of assessing DS as a distinct clinical phenotype regardless of the underlying aetiology.

This study had a few limitations. First, some bias could exist in the results of the questionnaire because the questionnaire was distributed to 50 IPISTs that had reported experience of working with many complex cases in our nationwide survey in 2021 [26], and the questionnaire response rate was 66%. Furthermore, requesting the submission of two ‘difficult to manage’ complex cases in each IPIST introduced a purposive selection bias. Second, the diagnosis may have been partially inaccurate because cases in these complex cases sometimes refuse detailed examinations. However, the IPIST is composed of at least one physician and two non‐physician professionals. Therefore, the diagnosis is considered to have some validity. Third, an assessment scale of DS, such as Environmental Cleanliness and Clutter Scale [39] was not used in this study. Therefore, overdiagnosis or underdiagnosis of DS may have occurred. However, we added the CIR to aid in the evaluation of the severity of DS. Finally, we did not perform statistical correction for multiple comparisons. We considered this study to be an exploratory investigation of the characteristics of DS. Therefore, we focused on avoiding the risk of β‐error rather than α‐error in this study with a small sample size.

In conclusion, the results of this study indicated that in this sample, many older people with DS exhibited self‐neglect and had limited family support. Many of them also had dementia. Our findings suggest that older people with DS and their families rarely consult appropriate services by themselves. We need to work with not only older people with DS but also their families who may have psychosocial problems. A multidisciplinary team with an outreach function may be useful for early intervention for older people with DS.

## Funding

This work was supported by the Ministry of Health, Labour and Welfare (MHLW) Program JPMH20CA2088 and the JSPS KAKENHI 21K21189 to DT and T25K025920 to MI.

## Disclosure

The authors have nothing to report.

## Ethics Statement

This study was approved by the Ethics Committees of Osaka University Hospital (Study Number 21353 (T1), Suita, Japan).

## Conflicts of Interest

The authors declare no conflicts of interest.

## Supporting information


**Figure S1:** Questionnaire about the complex cases in The Initial‐phase Intensive Support Team for Dementia (IPIST).

## Data Availability

The original contributions presented in the study are included in the article/Supporting Information; further inquiries can be directed to the corresponding author.
