# Supplementary material for: Characteristics of Older Adults With Domestic Squalor in Japan: A Cross‐Sectional Study
Source: Psychogeriatrics. 2026 Feb 25;26(2):e70150. doi: 10.1111/psyg.70150 (PMC12933695; doi:10.1111/psyg.70150)
Supplement: Supplementary file 1 — Figure S1: Questionnaire about the complex cases in The Initial‐phase Intensive Support Team for Dementia (IPIST). [file PSYG-26-0-s001.pdf]

Questionnaire about the complex cases in The Initial-phase Intensive Support Team for Dementia (IPIST)

☐agree ☐disagree

Local government **Prefectures** ________ **Municipality** ____________
Business entity ____________________

Older person receiving IPIST support **Age** (years) **Sex** ____ **Living condition** ____
Family living with case ____ **Care giver** ________
diagnosis ________________

Independence degree of daily living

Independence degree of daily living for older persons ____

Independence degree of daily living for patients with dementia ____

Reasons for the complexity

Reasons for the older person receiving IPIST support
(Multiple answers allowed)

☐ hallucinations/delusion ☐ irritability/aggression ☐ symptoms of disease
☐ violence ☐ drinking ☐ self-care
☐ driving ☐ taking medicine ☐ financial management

Caregivers' reasons
(Multiple answers allowed)

☐ absence ☐ old age ☐ exhaustion
☐ physical disease ☐ mental disease ☐ maltreatment
☐ lack of understanding ☐ poverty

Social reasons
(Multiple answers allowed)

☐ trash house ☐ problems with fire
☐ problems with neighbor ☐ police intervention

Other reasons
(Free-text comment)

☐

The first reason for difficulty among the above ____________________

The second reason for difficulty among the above ____________________

The third reason for difficulty among the above ____________________

People who experienced difficulties (Multiple answers allowed)

☐ Older person receiving IPIST support ☐ Care giver ☐ Supporter

Intention

Older person receiving IPIST support

Care giver

Supporter

History up to the start of support by IPIST

①First referral sources __________

②Whether through the Community Comprehensive Support Center__________

③Whether having family physician____

④Referral sources for IPIST__________

Detaled history of older person receiving IPIST support (Free-text comment, without personal information)

History after the sart of support by IPIST

①The need to see a specialist doctor __________

②The department of a specialist that this person has seen__________

③Experienced in psychiatric ca ☐ Mental Health Social Worke ☐ Nurse ☐ Occupational therapist ☐ Other

④Time from start of support to first medical examination__________

⑤Time from start of support to introduction of services__________

⑥Period from start of support to end of support __________

Detaled history about older person receiving IPIST support (Free-text comment, without personal information)

What was difficult or did not work?

How did you intervene in this person? What worked well?

Final opinion of the individual.
